# Influence of Chronic Kidney Disease on Platelet Reactivity Response to Clopidogrel and Ticagrelor

**DOI:** 10.3390/ijms27031359

**Published:** 2026-01-29

**Authors:** André Franci, Roberto Giraldez, Carlos Barbosa, Talia Dalçóquio, Paulo Genestreti, Aline Ferrari, Fernando Menezes, Remo Furtado, Danilo Sarti, Luciano Baracioli, José Nicolau

**Affiliations:** 1 Instituto do Coracao (InCor), Hospital das Clinicas HCFMUSP, Faculdade de Medicina, Universidade de Sao Paulo, Sao Paulo CEP 05403-000, Brazil; andre.franci@hsl.org.br (A.F.);; 2 Centro de Cardiologia, Hospital Sírio-Libanês, Sao Paulo CEP 01308-050, Brazil; 3 Hospital do Coracao do Brasil, Brasilia CEP 70390-000, Brazil; 4 Royal College of Surgeons in Ireland, University of Medicine and Heath Sciences–Stephen’s Green, D02 YN77 Dublin, Ireland

**Keywords:** platelet reactivity, coronary artery disease, chronic kidney disease, clopidogrel, ticagrelor

## Abstract

High platelet reactivity (HPR) in patients with coronary artery disease receiving P2Y12 inhibitors increases ischemic risk. Chronic kidney disease (CKD) is an established contributor to HPR during clopidogrel therapy. The objective of the study was to assess whether CKD influences platelet reactivity (PR) in patients treated with clopidogrel or ticagrelor. This double-blind, double-dummy study enrolled 106 stable patients more than one year after an acute coronary syndrome, with or without CKD. Participants were matched by age and sex and randomized to clopidogrel or ticagrelor. PR was measured using the VerifyNow™ P2Y12 assay, and HPR was defined as P2Y12 reaction units (PRU) ≥ 208. Median glomerular filtration rates were 80 mL/min/1.73 m^2^ in non-CKD patients and 41 mL/min/1.73 m^2^ in CKD patients (*p* < 0.01). Ticagrelor produced similarly low PR in both groups (36 vs. 35 PRU; *p* = 0.61). Clopidogrel resulted in a numerically higher PR in CKD patients (209 vs. 180 PRU; *p* = 0.07). The magnitude of PR reduction with ticagrelor relative to clopidogrel was greater in CKD patients (*p*-interaction = 0.09). HPR was markedly more common with clopidogrel, particularly in CKD (difference 37%; adjusted OR 4.42; *p* = 0.01). In conclusion, CKD significantly impairs clopidogrel responsiveness but does not affect ticagrelor, resulting in a greater relative advantage of ticagrelor in patients with CKD.

## 1. Introduction

Cardiovascular diseases (CVDs) stand as the leading cause of death globally, and ischemic heart disease (IHD) represents almost 50% of those fatal events. The prevalence of CVDs nearly doubled from 1990 to 2019 and deaths attributed to CVDs increased from 12.1 million to 18.6 million in this same period [1]. Chronic kidney disease (CKD) is recognized as an independent risk factor for the development and progression of coronary artery disease (CAD), and patients with CKD are more likely to die from CVDs than to progress to end-stage renal disease (ESRD) [2]. In patients hospitalized with an acute coronary syndrome (ACS), between 20 and 40% also have CKD [3]; on the other hand, in patients with a successful percutaneous coronary intervention (PCI), mostly following an ACS, one-year mortality was shown to be 5-fold higher in patients with moderate CKD and 12-fold higher in patients with severe CKD than in those with normal kidney function [4].

Despite ongoing debate regarding the optimal duration of dual antiplatelet therapy (DAPT) [5,6], it remains the cornerstone of treatment for patients with CAD undergoing PCI, whether in the setting of ACS or stable disease [7]. Patients with CAD taking P2Y12-inhibitors who maintain on-treatment high platelet reactivity (HPR) have an increased risk of ischemic cardiovascular events [8,9,10,11]. Moderate to severe CKD, defined as glomerular filtration rate (GFR) < 60 mL/min/1.73 m^2^, besides being a major risk factor for the occurrence of cardiovascular events, has also been associated with HPR in patients treated with clopidogrel [12,13]. Although CKD patients face a greater risk for both ischemic and bleeding events, the best DAPT regimen for these patients remains controversial, since they are frequently excluded from major clinical trials [14].

In a prespecified subanalysis from the PLATO trial, patients with CKD experienced a 23% relative risk reduction in the primary ischemic end point with ticagrelor compared with clopidogrel, whereas the reduction was only 10% among patients without CKD [15]. Although the *p*-value for interaction was non-significant (*p* = 0.13) when CKD was defined using the Cockcroft–Gault equation, it reached statistical significance (*p* = 0.03) when CKD was defined by the Modification of Diet in Renal Disease (MDRD) equation [15,16,17].

In spite of the fact that there are still conflicting data on the association of CKD with HPR in patients taking clopidogrel [18,19,20], a greater difference in platelet inhibition with ticagrelor over clopidogrel in patients with CKD could explain, at least partially, the apparent higher superiority of ticagrelor over clopidogrel demonstrated in the PLATO study within this population [21,22]. The present study was therefore focused on exploring the relationship between the antiplatelet effects of clopidogrel and ticagrelor in patients with and without CKD.

The main objective was to compare the inhibition of platelet aggregation (difference between clopidogrel and ticagrelor) evaluated by VerifyNow^TM^ P2Y12 (VNow) in patients with CAD, with and without CKD, undergoing treatment with ASA in combination with clopidogrel or ticagrelor. The comparisons were caried out considering 3 different analyses: (1) Delta of platelet reactivity (delta-VNow), which represents the difference between baseline and on-treatment P2Y12 reaction units (PRU) measured by VNow; (2) proportion of patients with HPR, defined as on-treatment platelet reactivity ≥ 208 PRU measured by VNow; and (3) percentage of platelet inhibition (%Inhib-VNow), calculated by the following formula: 100 × (Baseline PRU − On-treatment PRU)/Baseline PRU. Pre-specified secondary objective s included the comparison of platelet reactivity with Multiplate^®^ between the groups.

## 2. Results

**Figure 1:**
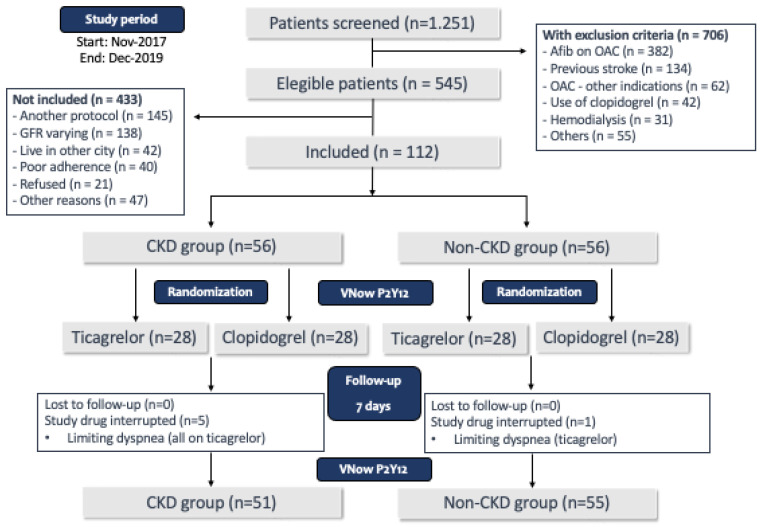
Study flow chart.

Figure 1 shows the study flow chart. As can be noticed, 1251 patients were initially screened, and 706 had at least one exclusion criteria for participation in the study. Of the 545 patients who met the inclusion criteria and did not have any exclusion criteria, many were already participating in other institutional protocols or had oscillations in creatinine values that prevented a clear and consistent classification regarding renal function. As planned, the study ended after 112 patients were included (56 in each group, non-CKD and CKD). There were no losses to follow-up, but five patients from the CKD group and one patient from the non-CKD group discontinued the study medication. All patients who discontinued the medication had been randomized to ticagrelor, and the reason for the discontinuation was limiting dyspnea. No other serious adverse events were reported. The 106 patients who tolerated the use of the study medication during the entire follow-up period were considered for the main objective analysis (51 patients in the CKD group and 55 patients in the non-CKD group).

Table 1 shows the baseline characteristics of the population according to the randomization to clopidogrel or ticagrelor. As expected, the groups were well balanced, without significant differences between them. Table 2 shows the same parameters but compares CKD and non-CKD patients. Patients from the CKD group had higher mean weight and lower utilization of angiotensin-converting enzyme inhibitor/angiotensin-II receptor blockers (ACEi/ARBs) in comparison with patients from the non-CKD group; additionally, the non-CKD group had higher hemoglobin and lower ultrasensitive C-reactive protein (usCRP) and triglyceride values compared with the CKD group.

### 2.1. Primary Objective: Comparison of Platelet Inhibition Measured by VNow Between Groups

**Figure 2:**
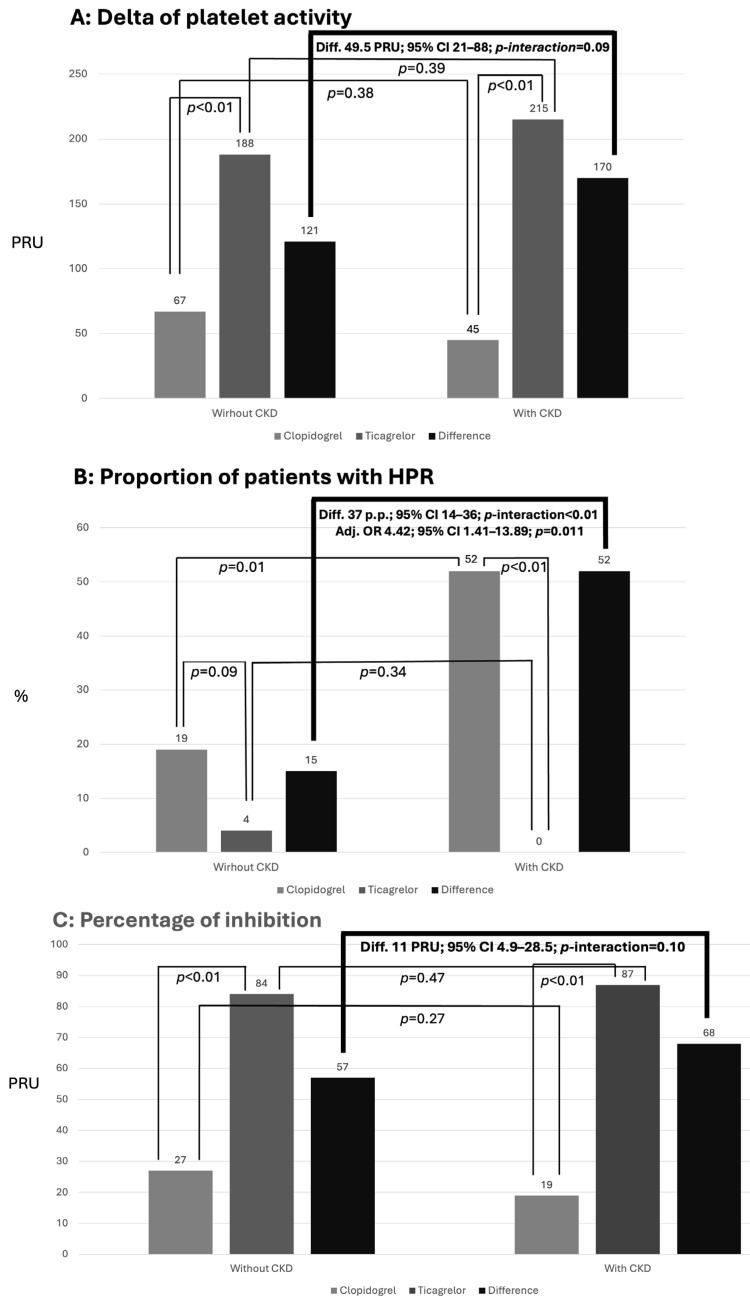
Platelet reactivity measured by VerifyNow^TM^ P2Y12 assay considering (**A**) the delta of platelet reactivity (on-treatment minus baseline), (**B**) proportion of patients with high platelet reactivity, and (**C**) % of platelet reactivity inhibition (see Table 3).

At the end of patient allocation, four groups were analyzed and compared for the primary endpoint: patients with CKD randomized to clopidogrel (CKD-C), patients with CKD randomized to ticagrelor (CKD-T), patients without CKD randomized to clopidogrel (non-CKD-C) and patients without CKD randomized to ticagrelor (non-CKD-T). As noted in Table 3 and Figure 2, baseline platelet aggregability measured by VNow was the same across all groups but, as expected, at the end of treatment, patients randomized to ticagrelor showed significantly lower levels of platelet aggregation compared to patients treated with clopidogrel, both in the non-CKD group (*p* < 0.01) as well as in the CKD group (*p* < 0.01). The same pattern was noted when analyzing the Delta-VNow and %Inib-VNow values, with significantly higher platelet inhibition of ticagrelor in comparison with clopidogrel both in non-CKD and CKD patients. Regarding HPR, there was no significant difference between clopidogrel and ticagrelor in the non-CKD population, but a *p*-value < 0.01 was observed for the CKD population. When analyzing the influence of the CKD in the results considering the “difference of the differences” [(clopidogrel minus ticagrelor in non-CKD) minus (clopidogrel minus ticagrelor in CKD)], a difference of 37 percentage pontis (p.p.) was observed, with a *p*-value < 0.01 for the interaction between clopidogrel, ticagrelor and presence or not of CKD.

### 2.2. Secondary Objective: Comparison of Platelet Inhibition Measured by Multiplate^®^ Between Groups

As can be seen in Table 4, no differences were observed between clopidogrel and ticagrelor in baseline platelet reactivity both in non-CKD and CKD groups; however, as demonstrated with VNow, after treatment patients randomized to ticagrelor showed significantly lower levels of platelet aggregation compared to patients treated with clopidogrel, both in the non-CKD (*p* = 0.01), as well as in the CKD group (*p* = 0.03). The delta-MP of patients on clopidogrel in comparison with ticagrelor was significantly lower in the CKD group (*p* < 0.01) but not in the non-CKD group (*p* = 0.09). Regarding the %Inhib-MP, ticagrelor was superior to clopidogrel both in the non-CKD group (*p* = 0.01) and in the CKD group (*p* < 0.01), and no significant differences between the groups were observed for HPR. Finally, in the “difference of the differences” a significant interaction was observed for the Delta-MP (*p* < 0.01), with a numerical superiority of ticagrelor over clopidogrel (*p*-interaction = 0.06).

## 3. Discussion

To the best of our knowledge, this is the largest randomized clinical study comparing the antiplatelet response to treatment with two P2Y12 inhibitors (clopidogrel and ticagrelor) in stable CAD patients with and without CKD. Our study suggests that the influence of CKD in the antiplatelet response is more pronounced in patients taking clopidogrel in comparison to those taking ticagrelor.

Meta-analysis including 73 studies analyzing the relationship between CKD and platelet reactivity concluded that the majority of the publications reported impaired platelet function in patients with CKD, but there was still a substantial number of studies showing unchanged or even increased platelet reactivity [23]. The majority of the studies analyzed the problem considering HPR, since many studies and meta-analyses reported an association of HPR with cardiovascular events [8,24,25,26], but we must recognize that conflicting data had also been published [27,28]. Different methodologies for assessing platelet aggregability, including different cut-off values for the definition of HPR, as well as the moment of carrying out this assessment and also the risk profile of the patients included in each of these studies, are some factors that could be related to the heterogeneity of the results [29,30].

In the same direction, pharmacodynamic studies have shown discordant results on the independent role of CKD as a determinant of HPR in patients taking clopidogrel, despite the fact that the results were unanimous in non-adjusted models [13]. In a cross-sectional observational study published by Angiolillo et al. analyzing patients with diabetes mellitus on DAPT (ASA + clopidogrel), those with moderate/severe CKD were more likely to have HPR, even after multiple adjustment for possible confounders [31]. A post hoc analysis from the ADAPT-DES registry compared the prevalence of HPR assessed by VNow in 8582 patients treated with DAPT (ASA + clopidogrel) after undergoing PCI [12]. In this population, HPR was more common among CKD compared to non-CKD patients and the prevalence of HPR increased in a graded fashion as renal function worsened. However, after multivariable adjustments, that association lost statistical significance, suggesting that confounding risk factors that were more prevalent in CKD patients could account for HPR in this population. Importantly, Li et al. showed that, although the relationship between HPR and major adverse cardiovascular events (MACE) was consistent across CKD strata without significant interaction between patients with or without CKD, adding platelet reactivity to GFR improved the area under the curve of the prediction model for MACE [32].

From the clinical standpoint, there is some indirect evidence from subgroup analysis of clinical trials that supports the results of pharmacodynamic studies on the impact of CKD on the antiplatelet response to clopidogrel. In a subanalysis of the CREDO trial, clopidogrel was associated with a significant reduction in the composite endpoint of death, myocardial infarction, and stroke in patients with normal kidney function (10.4% vs. 4.4%, HR = 0.42, 95% CI 0.26–0.69, *p* = 0.001) compared to placebo, but not in those with mild or moderate CKD [33]. In the CHARISMA trial, patients with diabetes mellitus and CKD who received clopidogrel had an increased cardiovascular and overall mortality compared with those assigned to placebo (HR = 1.856, 95% CI 1.106 to 3.116, *p*-interaction = 0.019) [34]. A metanalysis published by Palmer et al. with almost 10,000 patients concluded that DAPT (basically aspirin plus clopidogrel or IIb/IIIa inhibitors) in patients with CKD had little or no effect on reducing ischemic events such as myocardial infarction and cardiovascular mortality, but significantly increased bleeding [35]. On the other hand, the prespecified subgroup analysis from the PLATO trial published by James et al. showed a numerically larger superiority of ticagrelor over clopidogrel in patients with CKD in comparison to patients without CKD [15].

Our data on antiplatelet response and the prevalence of HPR suggest a stronger influence of CKD among patients treated with clopidogrel compared with ticagrelor, both in univariate analyses and after adjustment for confounding factors. This may explain, at least partially, the clinically proven superiority of ticagrelor over clopidogrel. Moreover, our interaction analyses involving clopidogrel, ticagrelor and CKD across different parameters and methods suggests that the superiority of ticagrelor over clopidogrel is more pronounced in patients with CKD, even though some of the comparisons did not reach statistical significance. Considering the superiority of ticagrelor over clopidogrel in a broad population with ACS, as demonstrated in PLATO [36], these pathophysiological results support previous publications suggesting the benefit of ticagrelor is larger in the high-risk population with CKD [15]. However, given the high prevalence of CKD among patients with ACS, and the heterogeneity of efficacy and safety results in this population [37], a dedicated, larger randomized clinical trial addressing this topic is advisable.

There are some limitations in our study that should be acknowledged. Firstly, we only included patients with chronic coronary disease who had at least 1 year since their last hospitalization for ACS, so these results may not apply to patients within the first year after an ACS event. Secondly, urinary albumin/creatinine was not collected, which might have altered the classification of some patients included in the non-CKD group based solely on estimated GFR. Thirdly, five patients in the ticagrelor group discontinued treatment because of dyspnea, which could have influenced our results, since there is evidence that the presence of dyspnea is associated with lower platelet reactivity [38]. Finally, the results on platelet aggregability with ticagrelor should not be interpreted as representative of other new antiplatelet drugs such as prasugrel or cangrelor, as there are many pharmacological differences between them, including non-antiplatelet pleiotropic effects.

## 4. Materials and Methods

### 4.1. Study Design and Patients

Stable patients followed at the Heart Institute of the Clinical Hospitals of the University of São Paulo Medical School with a history of ACS at least one year previous to the inclusion in the study were selected as cases (CKD) and controls (no CKD). Within each group—cases and controls—participants were randomized in a double-blind, double-dummy fashion to receive either ticagrelor or clopidogrel.

**Figure 3:**
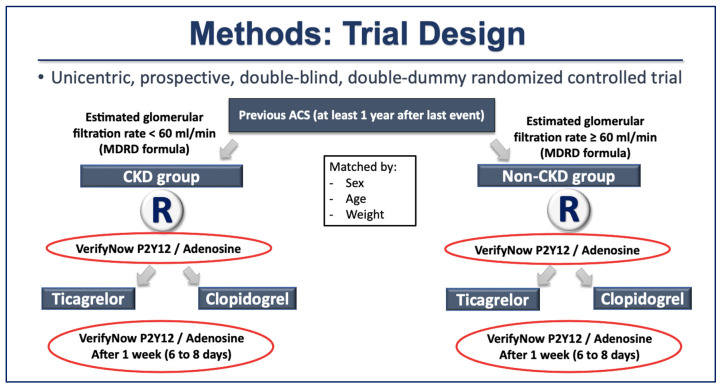
Study design diagram.

Patients 18 years or older, clinically stable, with previous ACS (at least 1 year) and atherosclerotic CAD, defined by coronary artery stenosis ≥50% in at least one major coronary artery documented by coronary angiography, were screened for inclusion in the study. They were divided into groups according to their GFR calculated by the MDRD formula. Patients with GFR < 60 mL/min/m^2^ were considered as the CKD group, those with GFR ≥ 60 mL/min/m^2^ were considered as the non-CKD group, and both groups were matched by age, sex, and weight. Those fulfilling the inclusion criteria and without any exclusion criteria were invited to participate and, after signing the informed consent form (ICF), were randomized in a 1:1 to treatment with clopidogrel 600 mg (loading dose) followed by 75 mg QD or ticagrelor 180 mg (loading dose) followed by 90 mg BID for 1 week (8 ± 2 days). The sequence for random allocation (generated by GraphPad Software, Boston, MA, USA, http://www.graphpad.com/quickcalcs/ (accessed in 18 June 2019), placebo production, and medication container fulfillment e numbering was made by the Pharmacy Division from the Hospital das Clinicas da Faculdade de Medicina da Universidade de Sao Paulo (HCFMUSP). An assistant physician from InCor/HCFMUSP was responsible for enrolling, explaining all steps in the study, and the final follow up medical visit; and a registered research nurse was responsible for the blood draws. The physician, nurse, and patients were all blinded to the medication to which the patients were randomized. The main exclusion criteria were history of previous ischemic or hemorrhagic stroke at any time; use of oral anticoagulants and/or DAPT and/or non-steroidal anti-inflammatory drugs in the last 30 days; formal indication for continuous use of proton pump inhibitors; known platelet dysfunction or platelet count < 100,000/µL or >450,000/µL; patient on dialysis; terminal illness with life expectancy less than 1 year; any known liver disease or coagulation disorder. One week (8 ± 2 days) post-randomization, patients were assessed for study drug adherence and any adverse events during the study. Figure 3 shows the study design diagram.

Phone contacts were available for all study participants to clarify any doubts or report possible adverse events during the study. This protocol is in accordance with the recommendations contained in the Declaration of Helsinki and was approved, together with the ICF, by the Scientific Committee of InCor/HCFMUSP (4086/14/066) and by the Research Ethics Committee from HC/FMUSP (CAAE 35079514.8.0000.0068). All volunteers were duly informed about the experimental protocol. All patients signed the ICF and the study protocol was registered on ClinicalTrials.gov (NCT03039205). This trial received funding from the Fundação de Amparo à Pesquisa do Estado de São Paulo (FAPESP), a public state agency for research support, under the award number 2014/01021-4.

### 4.2. Platelet Function Assessment

After randomization and before receiving the loading dose of the study drug, patients had their vital signs registered, followed by blood samples drawn for biochemical evaluation and platelet function testing. All blood samples were collected between 10 a.m. and 1 p.m., with 12 h fasting, by antecubital venipuncture with a 21 G needle and a tourniquet time less than 1 min. Platelet function testing was performed between 30 min and 2 h after blood draw. Platelet reactivity assessment was repeated after 8 ± 2 days from randomization. Platelet reactivity was primarily evaluated with the VerifyNow^TM^ P2Y12 assay (VNow), Werfen, Barcelona, Spain, a point-of-care turbidimetric-based system that measures platelet aggregation in whole blood by analyzing the degree of light penetration, with values expressed as PRU [39]. At the same timepoints, platelet reactivity was also assessed by Multiplate^®^ multiple electrode aggregometry (Roche Diagnostics International Ltd., Rotkreutz, Switzerland), a point-of-care platelet function analyzer that uses the principle of impedance aggregometry and expresses the results as the area under the curve (AUC) after stimulation with an ADP agonist.

### 4.3. Sample Size Calculation

Considering previously available data, platelet reactivity measured by VNow in non-CKD patients was 230 ± 70 PRU with clopidogrel and 50 ± 30 PRU with ticagrelor, resulting in a 180 PRU difference between both drugs [21,22]. Sample size was calculated considering that in patients with CKD, this difference would be 30% higher, since we expected that ticagrelor would maintain its antiplatelet effect, whereas the antiplatelet response to clopidogrel would be decreased in the presence of CKD. For a type I error of 0.01 and 90% power, 50 patients in each group should be included to test our hypothesis. Considering possible losses, we planned to include a total of 112 patients (56 in the CKD and 56 in the non-CKD group).

### 4.4. Statistical Analysis

For categorical variables, data is presented as absolute numbers and percentages. The Kolmogorov–Smirnov test was used to test the normality distribution of continuous variables, which are presented as mean ± standard deviation or median (25th and 75th percentiles), according to Gaussian or non-Gaussian distribution, respectively. Categorical variables were compared between groups using the chi-square test or Fisher’s exact test when indicated. For comparisons between proportions of paired samples, the McNemar test was used. For the comparison between independent samples, Student’s *t* test or Mann–Whitney were used, respectively, for variables with Gaussian or non-Gaussian distribution. For the comparison of continuous variables between paired samples, Student’s *t* test (if Gaussian distribution) or Wilcoxon test (if non-Gaussian distribution) were used. For the comparison of the differences between ticagrelor and clopidogrel within each group, Student’s *t* test (Gaussian) or Mann–Whitney U test (non-Gaussian) was applied, and for the analysis of the difference in the differences—that is, the difference between ticagrelor and clopidogrel in the CKD group minus the difference between ticagrelor and clopidogrel in the CKD group—the permutation test (5000 permutations) with 95% CI via stratified bootstrap (5000 resamples) was used. For the comparison of the difference between the proportion of patients with HPR in the ticagrelor and clopidogrel groups for patients with or without CKD the chi-square test or Fisher’s exact test (when indicated) was applied, and the permutation test (5000 permutations); 95% CI via stratified bootstrap (5000 resamples) was used to assess the difference of these differences. Finally, to investigate the variables that were independently associated with CKD, a stepwise logistic regression analysis was performed, considering CKD as the dependent variable and the following independent variables (those with a *p*-value < 0.10 in univariate analyses): HPR (or delta of platelet reactivity), body weight, history of hyperlipidemia, use of ACE inhibitors or angiotensin receptor blockers, C-reactive protein, hemoglobin, and triglycerides.

Additionally, we conducted complementary, non-parametric analyses in R Statistical Software (R Core Team, version 4.5.2, Vienna, Austria). Outcomes were defined for VNow as baseline PRU, on-treatment PRU, Δ PRU = baseline − on-treatment, percent inhibition = 100 × (baseline − on-treatment)/baseline [39], and high on-treatment platelet reactivity (HPR) using the prespecified cutoff PRU ≥ 208. For Multiplate ADP, analogous outcomes were computed for MP-ADP AUC, with HPR defined a priori as MP-ADP ≥ 46. Group summaries were reported as median (IQR). To assess treatment-by-CKD effect modification on robust location measures, we calculated crude difference-in-differences (ΔΔ) on medians for Δ PRU and % inhibition and on proportions for HPR across the 2 × 2 cells (non-CKD/CKD × clopidogrel/ticagrelor). The 95% confidence intervals for ΔΔ were obtained by stratified bootstrap resampling within each cell (5000 replicates). Two-sided *p*-values for ΔΔ were computed by permutation tests (5000 permutations) that randomly permuted the CKD factor while preserving cell sizes; significance was set at α = 0.05. These complementary R analyses were applied both to VNow and to Multiplate ADP outcomes and used the same per-protocol set as the main objective.

The statistical program used was SPSS (Statistical Package for Social Sciences), version 25, for MacOS and R. Results with a *p*-value lower than 0.05 two-tailed were considered statistically significant.

## 5. Conclusions

In conclusion, the presence of CKD negatively influences the antiplatelet response to clopidogrel, but not to ticagrelor, leading to a more pronounced superiority of ticagrelor over clopidogrel in patients with CKD than in those without CKD.

## Figures and Tables

**Table 1:** Baseline characteristics of the population according to randomization.

	Clopidogrel n = 56	Ticagrelor n = 50	*p*-Value
Age—years ± SD	71.7 ± 4.7	70.7 ± 4.4	0.27
Female sex—n (%)	16 (28.6)	13 (26.0)	0.77
Weight—Kg ± SD	74.3 ± 11.3	73.1 ± 11.0	0.42
BMI—Kg/m^2^ ± SD	27.6 ± 3.7	27.6 ± 3.3	0.99
HTN—n (%)	49 (87.5)	48 (96.0)	0.12
Diabetes mellitus—n (%)	27 (48.2)	25 (50.0)	0.85
Hyperlipidemia—n (%)	50 (89.3)	41 (82.0)	0.28
CKD—n (%)	28 (50.0)	23 (46.0)	0.68
Smokers—n (%)	3 (5.4)	7 (14.0)	0.13
Previous AMI—n (%)	55 (98.2)	49 (98.0)	0.94
Previous PCI—n (%)	41 (73.2)	34 (68.0)	0.56
Previous CABG—n (%)	19 (33.9)	18 (36.0)	0.82
Coffee drinkers—n (%)	48 (85.7)	46 (92.0)	0.37
**Medications**			
ASA—n (%)	56 (100)	50 (100)	1.00
Statin—n (%)	55 (98.2)	50 (100)	0.34
Betablockers—n (%)	52 (92.9)	49 (98.0)	0.21
ACEi/ARBs—n (%)	45 (80.4)	38 (76.0)	0.59
CCB—n (%)	20 (35.7)	17 (34.0)	0.85
OHA—n (%)	25 (44.6)	29 (58.0)	0.17
Insulin—n (%)	10 (17.9)	6 (12.0)	0.40
PPI—n (%)	22 (39.3)	18 (36.0)	0.73
**Laboratory tests**			
VNow—PRU ± SD	251 ± 39	245 ± 41	0.70
Multiplate^®^—AUC ± SD	71 ± 20	76 ± 23	0.46
Hemoglobin—g/dL (Q1–Q3)	13.7 (12.9–14.5)	14.0 (12.9–15.1)	0.46
WBC count × 10^3^/mm^3^ (Q1–Q3)	7.2 (5.7–8.1)	7.8 (6.5–8.7)	0.31
Platelet count × 10^3^/mm^3^ (Q1–Q3)	196 (165–234)	202 (175–228)	0.93
IPF—% (Q1–Q3)	5.1 (3.1–6.7)	4.4 (3.2–7.4)	0.99
PT—seconds (Q1–Q3)	11.4 (11.0–11.9)	11.5 (11.0–12.1)	0.83
aPTT—seconds (Q1–Q3)	28.0 (26.1–30.8)	28.3 (26.0–30.9)	0.47
usCRP—mg/L (Q1–Q3)	1.12 (0.50–5.60)	1.21 (0.71–3.40)	0.25
Urea—mg/dL (Q1–Q3)	44 (37–62)	49 (31–60)	0.81
Creatinine—mg/dL (Q1–Q3)	1.13 (0.98–1.70)	1.07 (0.90–1.64)	0.49
GFR MDRD—mL/min (Q1–Q3)	57 (39–78)	67 (42–88)	0.52
Fasting glucose—mg/dL (Q1–Q3)	103 (96–127)	112 (98–122)	0.33
HbA1C—% (Q1–Q3)	6.0 (5.7–6.6)	6.2 (5.7–6.7)	0.82
Lp(a)—mg/dL (Q1–Q3)	29 (12–64)	19 (7–55)	0.16
TC—mg/dL (Q1–Q3)	150 (131–171)	141 (123–166)	0.30
LDL-C—mg/dL (Q1–Q3)	78 (68–105)	74 (58–89)	0.22
HDL-C—mg/dL (Q1–Q3)	44 (38–52)	42 (37–50)	0.25
Non-HDL-C—mg/dL (Q1–Q3)	106 (86–129)	99 (82–111)	0.49
TG—mg/dL (Q1–Q3)	114 (75–153)	112 (77–169)	0.50

SD: standard deviation; CKD: chronic kidney disease; BMI: body mass index; HTN: arterial hypertension; AMI: acute myocardial infarction; PCI: percutaneous coronary intervention; CABG: coronary artery bypass graft surgery; ASA: acetylsalicylic acid; ACEi: angiotensin-converting enzyme inhibitors; ARBs: angiotensin-II receptor blockers; CCB: calcium channel blockers; OHA: oral hypoglycemic agents; PPI: proton pump inhibitors. VNow: VerifyNowTM P2Y12 assay; PRU: P2Y12 reaction units; AUC: area under the curve; WBC: white blood cell; IPF: immature platelet fraction; PT: prothrombin time; aPTT: activated partial thromboplastin time; usCRP: ultrasensitive C-reactive protein; GFR MDRD: glomerular filtration rate estimated by the MDRD formula; HbA1C: glycated hemoglobin; Lp(a): lipoprotein (a); TC: total cholesterol; LDL-C: low-density lipoprotein cholesterol; HDL-C: high-density lipoprotein cholesterol; TG: triglycerides.

**Table 2:** Baseline characteristics of the population in non-CKD and CKD groups.

	Non-CKD n = 55	CKD n = 51	*p*-Value
Age—years ± SD	71.1 ± 4.6	71.4 ± 4.6	0.75
Female sex—n (%)	16 (29.1)	13 (25.5)	0.68
Weight—kg ± SD	71.9 ± 11.5	76.8 ± 11.6	0.02
BMI—Kg/m^2^ ± SD	27.6 ± 3.7	27.7 ± 3.5	0.77
HTN—n (%)	50 (90.9)	47 (92.2)	0.82
Diabetes mellitus—n (%)	23 (41.8)	29 (56.9)	0.12
Hyperlipidemia—n (%)	44 (80.0)	47 (92.2)	0.07
Smokers—n (%)	7 (12.7)	3 (5.9)	0.23
Previous AMI—n (%)	53 (96.4)	51 (100)	0.17
Previous PCI—n (%)	39 (70.9)	36 (70.6)	0.97
Previous CABG—n (%)	19 (34.5)	18 (35.3)	0.97
Coffee drinkers—n (%)	51 (92.7)	43 (84.3)	0.17
**Medications**			
ASA—n (%)	55 (100)	51 (100)	1.00
Statin—n (%)	54 (98.2)	51 (100)	0.33
Betablockers—n (%)	52 (94.5)	49 (96.1)	0.71
ACEi/ARBs—n (%)	50 (90.9)	33 (64.7)	<0.01
CCB—n (%)	17 (30.9)	20 (39.2)	0.37
OHA—n (%)	28 (50.9)	26 (51.0)	0.99
Insulin—n (%)	9 (16.4)	7 (13.7)	0.70
PPI—n (%)	24 (43.6)	16 (31.4)	0.19
**Laboratory tests**			
VNow PRU ± SD	242 ± 36	251 ± 41	0.08
Multiplate^®^ AUC ± SD	74 ± 20	72 ± 23	0.57
Hemoglobin—g/dL (Q1–Q3)	14.1 (13.4–15.4)	13.9 (12.6–14.4)	0.03
WBC count × 10^3^/mm^3^ (Q1–Q3)	7.3 (6.1–8.5)	7.3 (6.0–8.5)	0.81
Platelet count × 10^3^/mm^3^ (Q1–Q3)	194 (172–225)	197 (161–231)	0.87
IPF—% (Q1–Q3)	4.7 (3.4–7.2)	4.1 (3.0–6.5)	0.40
PT—seconds (Q1–Q3)	11.5 (11.1–12.0)	11.4 (10.8–11.9)	0.27
aPTT—seconds (Q1–Q3)	28 (26–30.7)	28.5 (26–31.7)	0.35
usCRP—mg/L (Q1–Q3)	0.77 (0.35–2.22)	1.54 (0.85–5.62)	<0.01
Urea—mg/dL (Q1–Q3)	37 (29–44)	58 (47–74)	<0.01
Creatinine—mg/dL (Q1–Q3)	0.97 (0.84–1.07)	1.69 (1.30–1.97)	<0.01
GFR MDRD—mL/min (Q1–Q3)	80 (68–94)	41 (35–52)	<0.01
Fasting glucose—mg/dL (IQR)	108 (96–118)	106 (93–132)	0.91
HbA1C—% (Q1–Q3)	6.1 (5.7–6.6)	6.1 (5.7–6.7)	0.69
Lp(a)—mg/dL (Q1–Q3)	23 (10–46)	32 (10–64)	0.29
TC—mg/dL (Q1–Q3)	148 (125–166)	144 (129–172)	0.66
LDL-C—mg/dL (Q1–Q3)	78 (59–94)	75 (60–98)	0.78
HDL-C—mg/dL (Q1–Q3)	42 (37–50)	43 (36–50)	0.93
Non-HDL-C—mg/dL (Q1–Q3)	100 (79–124)	102 (84–126)	0.62
TG—mg/dL (Q1–Q3)	98 (73–132)	134 (77–181)	0.02

SD: standard deviation; CKD: chronic kidney disease; BMI: body mass index; HTN: arterial hypertension; AMI: acute myocardial infarction; PCI: percutaneous coronary intervention; CABG: coronary artery bypass graft surgery; ASA: acetylsalicylic acid; ACEi: angiotensin-converting enzyme inhibitors; ARBs: angiotensin-II receptor blockers; CCB: calcium channel blockers; OHA: oral hypoglycemic agents; PPI: proton pump inhibitors. VNow: VerifyNow^TM^ P2Y12 assay; PRU: P2Y12 reaction units; AUC: area under the curve; WBC: white blood cell; IPF: immature platelet fraction; PT: prothrombin time; aPTT: activated partial thromboplastin time; usCRP: ultrasensitive C-reactive protein; GFR MDRD: glomerular filtration rate estimated by the MDRD formula; HbA1C: glycated hemoglobin; Lp(a): lipoprotein (a); TC: total cholesterol; LDL-C: low-density lipoprotein cholesterol; HDL-C: high-density lipoprotein cholesterol; TG: triglycerides.

**Table 3:** Platelet reactivity measured by VerifyNow^TM^ P2Y12 assay.

	Non-CKD-C (n = 27)	Non-CKD-T (n = 26)	*p*-Value (non-CKD)	CKD-C (n = 29)	CKD-T (n = 23)	*p*-Value (CKD)	Diff-of-Diff (95% CI)	*p*-Value (ΔΔ)
Baseline VNow, PRU (Q1–Q3)	245 (217–267)	236 (213–266)	0.908	266 (220–284)	256 (229–274)	0.513	—	—
On-treatment VNow, PRU (Q1–Q3)	180 * (106–198)	36 ** (24–55)	<0.01	209 (176–241)	35 (9–65)	<0.01	—	—
HPR—n (%)	5 (19)	1 (4)	0.09	15 (52)	0 (0)	<0.01	−37.1 p.p. (−61.6; −11.9)	<0.01
Delta-VNow, PRU (Q1–Q3)	67 (26–114)	188 (174–229)	<0.01	45 (24–90)	215 (168–239)	<0.01	49.5 (21.0; 88.0)	0.09
%Inhib-VNow, PRU (Q1–Q3)	27 (14–53)	84 (79–90)	<0.01	19 (8–33)	87 (73–96)	<0.01	10.8 (4.9; 28.5)	0.10

Non-CKD-C: patients without CKD randomized to clopidogrel; Non-CKD-T: patients without CKD randomized to ticagrelor; CKD-C: patients with CKD randomized to clopidogrel; CKD-T: patients with CKD randomized to ticagrelor; VNow: VerifyNow^TM^ P2Y12 assay; PRU: P2Y12 reaction units; Delta-VNow: the difference between baseline and on-treatment PRU measured by VNow; %Inhib-VNow: percentage of platelet inhibition, calculated by the following formula: 100 × (Baseline PRU − On-treatment PRU)/Baseline PRU; HPR: high platelet reactivity, defined as on-treatment platelet reactivity ≥ 208 PRU measured by VerifyNow^TM^. * *p* = 0.07 vs. CKD-C; ** *p* = 0.61 vs. CKD-T.

**Table 4:** Platelet reactivity measured by Multiplate^®^ assay.

	Non-CKD-C (n = 24)	Non-CKD-T (n = 22)	*p*-Value (non-CKD)	CKD-C (n = 28)	CKD-T (n = 21)	*p*-Value (CKD)	Diff-of-Diff (95% CI)	*p*-Interaction (ΔΔ)
Baseline Multiplate, AUC (Q1–Q3)	76 (60–84)	70 (58–95)	0.621	64 (51–88)	80 (65–92)	0.146	—	—
On-treatment Multiplate, AUC (Q1–Q3)	38 * (32–46)	28 ** (22–43)	0.010	34 (25–62)	25 (20–33)	0.034	—	—
HPR—n (%)	6 (25)	2 (9)	0.247	11 (39)	4 (19)	0.210	−4.3 p.p. (−36.9; 29.8)	0.784
Delta-Multiplate, AUC (Q1–Q3)	32 (25–42)	37 (33–55)	0.088	18 (8–32)	49 (33–59)	<0.01	27.0 (2.0; 39.5)	<0.01
%Inhib-Multiplate (Q1–Q3)	47 (37–53)	56 (51–71)	0.013	37 (11–56)	64 (48–75)	<0.01	18.1 (−5.7; 39.4)	0.056

Non-CKD-C: patients without CKD randomized to clopidogrel; Non-CKD-T: patients without CKD randomized to ticagrelor; CKD-C: patients with CKD randomized to clopidogrel; CKD-T: patients with CKD randomized to ticagrelor; %Inhib-Multiplate: percentage of platelet inhibition, calculated by the following formula: 100 × (Baseline AUC − On-treatment AUC)/Baseline AUC; HPR: high platelet reactivity, defined as on-treatment platelet reactivity ≥ 208 PRU measured by VerifyNowTM. * *p* = 0.59 vs. CKD-C; ** *p* = 0.35 vs. CKD-T.

## Data Availability

The original contributions presented in this study are included in the article. Further inquiries can be directed to the corresponding authors.

## Abbreviations

The following abbreviations are used in this manuscript:

ACS	Acute coronary syndrome
CAD	Coronary artery disease
CKD	Chronic kidney disease
CVDs	Cardiovascular diseases
DAPT	Dual antiplatelet therapy
ESRD	End-stage renal disease
GFR	Glomerular filtration rate
HPR	High platelet reactivity
ICF	Informed consent form
IHD	Ischemic heart disease
MDRD	Modification of Diet in Renal Disease
PCI	Percutaneous coronary intervention
PRU	P2Y12 reaction units
VNOW	VerifyNow^TM^ P2Y12 assay
